# Will Auranofin Become a Golden New Treatment Against COVID-19?

**DOI:** 10.3389/fimmu.2021.683694

**Published:** 2021-09-22

**Authors:** Karine Sonzogni-Desautels, Momar Ndao

**Affiliations:** ^1^Infectious Diseases and Immunity in Global Health Program, Research Institute of the McGill University Health Centre, Montreal, QC, Canada; ^2^Department of Microbiology and Immunology, Faculty of Medicine and Health Sciences, McGill University, Montreal, QC, Canada; ^3^National Reference Centre for Parasitology, Research Institute of the McGill University Health Centre, Montreal, QC, Canada

**Keywords:** auranofin, gold, COVID-19, coronavirus, SARS-CoV-2, thioredoxin, cytokine storm, IL-6

## Abstract

Auranofin is an FDA-approved disease-modifying anti-rheumatic drug that has been used for decades for treatment of rheumatoid arthritis. This gold(I) compound has anti-inflammatory properties because it reduces IL-6 expression *via* inhibition of the NF-κB-IL-6-STAT3 signaling pathway. Also, by inhibiting redox enzymes such as thioredoxin reductase, auranofin increases cellular oxidative stress and promotes apoptosis. Auranofin also possesses antiviral properties. Recently, it was reported that auranofin reduced by 95% SARS-CoV-2 RNA in infected human cells *in vitro* and decreased SARS-CoV-2-induced cytokine expression, including IL-6. During SARS-CoV-2 infection, a cytokine storm involving IL-6 increases severity of illness and worsens prognosis. Therefore, auranofin could, in our point of view, reduce pathology due to SARS-CoV-2-induced IL-6. COVID-19 is a rapidly-evolving respiratory disease now distributed worldwide. Strikingly high numbers of new COVID-19 cases are reported daily. We have begun a race to vaccinate people, but due to the complex logistics of this effort, the virus will continue to spread before all humans can be immunized, and new variants that may be less well contained by current vaccines are of concern. The COVID-19 pandemic has overwhelmed health care systems and new treatments to reduce mortality are urgently needed. We encourage to further evaluate the potential of auranofin in the treatment of COVID-19 *in vitro* and in animal models of SARS-CoV-2 infection and, if preliminary data are promising, in clinical trials with COVID-19 patients. In our opinion, auranofin has the potential to become a valuable addition to available therapies in this pandemic.

## Introduction

COVID-19, caused by the coronavirus SARS-CoV-2, has infected, as of March 7^th^ 2021, more than 116 millions of people and caused more than 2.5 million deaths worldwide due to sustained human-to-human transmission (1). The number of new COVID-19 cases has recently skyrocketed in many countries, particularly in the Americas and Europe (1). Many countries implemented regional or nationwide lockdown policies, curfews or stay-at-home orders to attempt to contain the spread of the virus. While vaccines targeting SARS-CoV-2 are now available, the appearance of more contagious new SARS-CoV-2 variants is a serious threat (2). If vaccine efficacy is reduced against emerging variants, all ground gained during the past year could be lost and SARS-CoV-2 could resurge again (2). We must also take into consideration the reluctance of some people to be vaccinated, and the time needed to immunize whole communities, worldwide. All these factors will leave part of the population non-immune, at least for some time. Moreover, immunity from natural infection or vaccination may not last for years and humanity would not be protected from a resurgence of SARS-CoV-2 or a SARS-like coronavirus in the future. For these reasons, there is an urgent need to find new treatments to significantly reduce COVID-19-related pathogenicity and mortality. That being said, discovering and developing a new drug as well as testing its safety is an expensive and time-consuming endeavor. On the contrary, repurposing an approved drug is more affordable and time efficient, which constitute important factors during a pandemic. However, repurposed drugs should be thoroughly evaluated *in vitro*, in animal models and clinical trials before being recommended in the treatment of COVID-19 patients. So far, clinical management of COVID-19 patients was based on supportive care while several repurposed drugs are being tested in clinical trials (3).

## Clinical Trials and Immunomodulatory Treatments

Clinical trials tested, among others, antiviral (e.g. remdesivir), anti-parasitic (e.g. ivermectin), anti-inflammatory (e.g. glucocorticoids) and antibody (e.g. tocilizumab) therapies to treat COVID-19 (4). In a randomized trial, remdesivir, a promising antiviral drug, shortened the time to recovery compared to placebo in COVID-19 patients (5). However, treatment with an antiviral drug alone is not sufficient to prevent mortality in all patients (5, 6). It was suggested that remdesivir therapy could show improved efficacy if administered before the inflammatory phase of COVID-19; this hypothesis would benefit further investigation (4, 6, 7). Recently, ivermectin, a macrocyclic lactone with broad-spectrum antiparasitic action, was considered promising in the treatment of COVID-19 due to its anti-SARS-CoV-2 effects *in vitro* (8). Ivermectin has known antiviral properties against several viruses such as yellow fever virus, dengue virus and chikungunya virus (9, 10). But, simulations based on pharmacokinetic studies performed in healthy volunteers predicted that ivermectin is unlikely to reach concentrations in lungs needed for anti-SARS-CoV-2 action, even if 10 times the approved dose is administered orally (11). At the latter dose, the maximum plasma concentrations would be one order of magnitude lower than the *in vitro* IC_50_ of ivermectin against SARS-CoV-2 (8, 12). Clinical trials are being performed and results will determine the potential of ivermectin in the treatment of COVID-19.

The COVID-19-associated inflammatory cytokine storm is at least partly responsible for increased severity of illness and mortality (13). COVID-19 patients could then benefit from the anti-inflammatory activity of corticosteroids during the inflammatory phase of the disease (3, 7). Preliminary studies showed that low-dose corticosteroids do not delay viral clearance (14) and that corticosteroids can improve survival of critically ill patients with COVID-19 (3, 7). Particularly, the Randomized Evaluation of COVID-19 Therapy (RECOVERY) trial of dexamethasone showed that this glucocorticoid reduced mortality in hospitalized patients receiving respiratory support (invasive mechanical ventilation or oxygen alone) (15). Based on data from this clinical trial, the Panel of the US National Institutes of Health (NIH) now recommends administration of dexamethasone to COVID-19 patients requiring supplemental oxygen (16).

Due to the major role of IL-6 in the COVID-19-associated inflammatory cytokine storm (13, 17), IL-6 inhibitors are attractive therapeutic options and several clinical studies evaluated or are ongoing to evaluate their potential to improve outcome during COVID-19 (18–21). A comprehensive review of ended and ongoing clinical trials on IL-6 inhibitors was already published (21). Tocilizumab is a monoclonal antibody against IL-6 receptor used in the treatment of rheumatoid arthritis and is, by far, the IL-6 inhibitor most studied as a potential therapy against COVID-19 (21). The promising role of tocilizumab was first revealed in anecdotal reports and small scale clinical studies. As examples, some reports showed that tocilizumab significantly reduced COVID-19-associated inflammatory response and prevented rapid clinical deterioration of COVID-19 patients with severe pneumonitis (18). Some clinical trials reported that treatment with tocilizumab leads to improvement of oxygenation and reduction of risk of mechanical ventilation and mortality (21). In the EMPACTA clinical trial, hospitalized COVID-19 patients not receiving mechanical ventilation, tocilizumab reduced the likelihood of progression to mechanical ventilation or death (22). However, in the EMPACTA clinical trial, mortality by day 28 was similar in patients treated with tocilizumab compared to patients treated with placebo (22). Other studies also showed no clinical improvement or no reduction in mortality (e. g. randomized controlled COVACTA trial) (21). Sarilumab is another anti-IL-6 receptor antibody used in the treatment of rheumatoid arthritis (18). Like tocilizumab, sarilumab is sometimes, but not always, associated with clinical improvement in COVID-19 patients (21). In contrast with tocilizumab and sarilumab, siltuximab is a human–murine chimeric monoclonal antibody that binds soluble forms of human IL-6 (19). Several clinical trials using tocilizumab, sarilumab or siltuximab are ongoing; their results will provide an important insight on the role of these IL-6 inhibitors in the treatment of COVID-19.

While IL-6 inhibitors are promising in the treatment of COVID-19, lack of efficacy of IL-6 inhibitors in some clinical trials advice against their use alone in COVID-19 patients. Therefore, combination therapies with other immunomodulatory molecules are of immediate interest (20, 21). As an example, in the ongoing COV-AID clinical trial, COVID-19 patients received in addition to standard care either tocilizumab, siltuximab or anakinra (anti-IL-1 binding the IL-1 receptor) or a combination of tocilizumab with anakinra or siltuximab with anakinra (20). Results of these clinical trials will define the impact of combination therapies in the outcome of COVID-19. Moreover, the combination of tocilizumab with corticosteroids already showed significant clinical improvement. The two largest randomized controlled trials on tocilizumab, named ‘Randomized, Embedded, Multifactorial Adaptive Platform Trial for Community- Acquired Pneumonia (REMAP-CAP)’ and ‘Randomized Evaluation of COVID-19 Therapy (RECOVERY)’, reported a reduced mortality following tocilizumab treatment in COVID-19 patients. The REMAP-CAP trial was a large international clinical trial enrolling critically ill hospitalized COVID-19 patients within 24 hours of intensive care unit level care (23). Clinical improvement in the REMAP-CAP trial was measured by median number of respiratory or cardiovascular organ support–free days, this number was 10 for COVID-19 patients treated with tocilizumab with standard of care (SOC) compared to 0 for control COVID-19 patients receiving only SOC (23). Also, tocilizumab treatment with SOC reduced mortality in the REMAP-CAP trial compared to SOC alone (23). During the time the REMAP-CAP trial was conducted, glucocorticoids were recommended in the SOC following the publication of the data of other clinical trials such as the REMAP-CAP on hydrocortisone (24) and the RECOVERY trial on dexamethasone (15). Therefore, the combination of tocilizumab with glucocorticoids as part of the SOC probably improved the results of the REMAP-CAP trial (23). In the RECOVERY trial on tocilizumab, hospitalized patients with severe or critical COVID-19 with hypoxia and high C-reactive protein levels were enrolled; 82% of enrolled COVID-19 patients also received systemic corticosteroids (25). Tocilizumab decreased mortality and increased chances to be discharged from hospital within 28 days (25). Also, among patients not under invasive mechanical ventilation at baseline, tocilizumab reduced the risk of invasive mechanical ventilation and death (25). The RECOVERY Collaborative Group therefore concluded that the benefits of tocilizumab were additional to the benefits of systemic corticosteroids (25). Consequently, the Panel of the NIH provides the following recommendations on their website (26): ‘The Panel recommends using ‘tocilizumab in combination with dexamethasone in certain hospitalized patients who are exhibiting rapid respiratory decompensation due to COVID-19’; however ‘The Panel recommends against the use of anti-IL-6 monoclonal antibody therapy (i.e., siltuximab) for the treatment of COVID-19, except in a clinical trial’ (26).

Supportive therapies for clinical management of severe COVID-19 are administered in hospital settings with close monitoring of patients. Effectively, COVID-19 is a rapidly-evolving disease and severe cases need to be hospitalized and treated under meticulous monitoring. However, an oral treatment that can be prescribed to patients with mild to moderate symptoms would help them recover at home while reducing viral replication and environmental contamination, therefore diminishing spread of the virus in the household. For patients, such therapy would reduce their risk of developing severe clinical symptoms and requiring hospitalization. For communities, such treatment would lower pressure on the health care system. The threat of a marked surge of COVID-19 cases that would overwhelm the health care system is a sword of Damocles hanging over us. If the pandemic worsens and hospitals overflow, prioritization of access to intensive care would need to be established. In that catastrophic scenario, an oral treatment could offer an alternative therapy for patients not requiring intensive care such as mechanical ventilation.

An oral drug with anti-inflammatory and anti-SARS-CoV-2 properties, that is already FDA-approved with a known toxicity profile would be a promising candidate in the treatment of COVID-19. Auranofin is FDA-approved and well tolerated in humans, based on decades of use for treatment of rheumatoid arthritis (RA) (27). Auranofin can be administered orally and its pharmacokinetics, pharmacodynamics and adverse effects have been described (28, 29). RA is an autoimmune disease causing inflammation, pain and swelling in articulations (30). While the mechanism by which auranofin reduces inflammation during RA is not fully understood, it is reported that auranofin can decrease expression of some pro-inflammatory cytokines (30). In fact, peripheral blood monocytes of RA patients treated with auranofin have lower basal and lipopolysaccharide-stimulated IL-6 productions compared to untreated RA patients (31). Also, expression of macrophage-derived IL-6, which is abundant in rheumatoid synovium, is also reduced by auranofin treatment *in vitro* (30). By its action on the redox milieu, *via* inhibition of redox enzymes such as thioredoxin reductase, auranofin also possesses anticancer, antiparasitic, antibacterial and antiviral properties which were reviewed elsewhere (32). In human cells infected with SARS-CoV-2, auranofin inhibited viral replication and markedly decrease expression of proteins of the inflammatory response, including IL-6 (33). If further investigations *in vitro* and in animal models of SARS-CoV-2 infection validate the anti-COVID-19 effects of auranofin, this gold compound would become a promising candidate in the treatment of COVID-19.

## Mechanisms of Action of Auranofin

**Figure 1:**
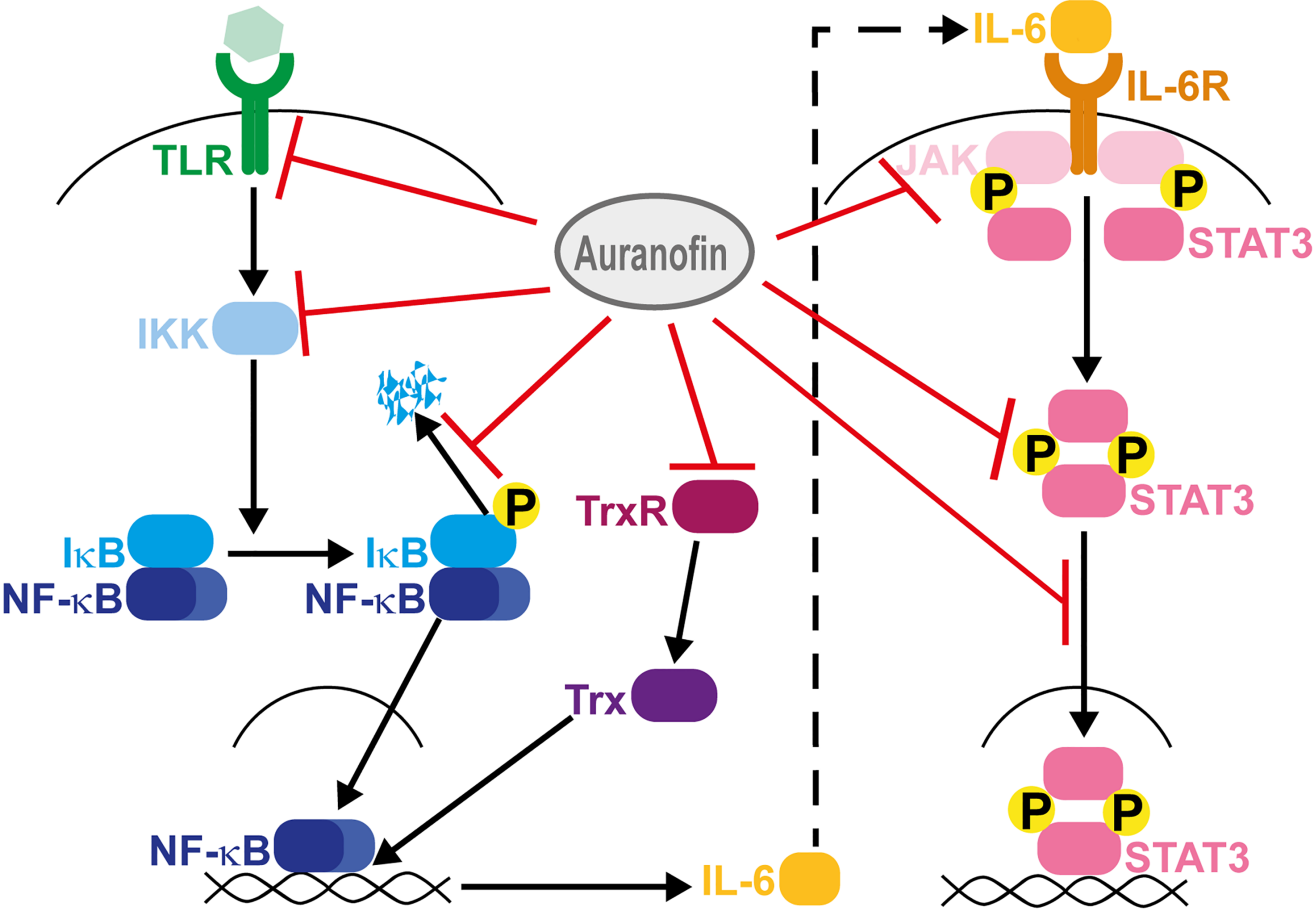
Mechanisms of action of auranofin. Auranofin inhibits dimerization of toll-like receptor (TLR) 4, activation of IκB kinase (IKK), degradation of IκB, IL-6-induced phosphorylation of Janus kinase 1 (JAK1) and signal transducer and activator of transcription 3 (STAT3), and STAT3 translocation to the nucleus. In addition, auranofin inhibits thioredoxin reductase (TrxR) and, consequently, TrxR-dependent activation of thioredoxin (Trx); auranofin therefore inhibits the Trx-induced promotion of NF-κB transactivation.

To understand the potential of auranofin for the treatment of COVID-19, knowledge of its mechanisms of action is necessary. Manipulating the redox milieu by inhibiting redox enzymes is the main mechanism of action of auranofin (27). Auranofin is a potent inhibitor of thioredoxin reductase, an enzyme that minimizes oxidative stress and promotes cell survival (27). Disruption of redox homeostasis by auranofin can therefore lead to redox-sensitive apoptosis (27). Thioredoxin reductase also regulates the transactivation of NF-κB, a transcription factor involved in inflammation and cell survival (34). Inhibition of thioredoxin reductase prevents NF-κB DNA binding and NF-κB-dependent gene expression (34). From previous studies on SARS-CoV, we predict that high IL-6 expression during COVID-19 cytokine storm is mediated by NF-κB (35). In fact, the SARS-CoV viral spike protein and nucleocapsid protein promote NF-κB-dependent IL-6 expression (13, 35). Strong NF-κB activation induces SARS-CoV-mediated lung inflammatory immunopathology and inhibition of NF-κB decreases mortality in SARS-CoV infected mice (13). Auranofin also inhibits homodimerization of TLR4 and TLR4-mediated activation of NF-κB (36). *In silico* studies recently showed that toll-like receptor (TLR) 4 could have a crucial role in SARS-CoV-2-induced inflammatory responses and that TLR4-antagonists are promising therapeutic candidates (37). Moreover, other TLRs are targeted by auranofin, including TLR3 (38), which is involved during SARS-CoV-2 infection (13). Auranofin also prevents activation of IκB kinase (IKK) which initiates phosphorylation of IκB and promotes NF-κB activity (39). In addition, auranofin suppresses the degradation of inhibitory IκB proteins associated with NF-κB (39). Equally important, auranofin inhibits IL-6-induced phosphorylation of Janus kinase 1 (JAK1) and signal transducer and activator of transcription 3 (STAT3) (40). Moreover, due to its inhibitory effect on STAT3 translocation to the nucleus, auranofin blocks expression of STAT3-regulated genes (40). Thus, auranofin inhibits several steps in the NF-κB-IL-6-STAT3 signaling pathway (**Figure 1**).

Because auranofin acts upstream and downstream of NF-κB-dependent IL-6 expression, both IL-6 secretion and STAT3-derived IL-6 action are inhibited (39, 40). As an important proinflammatory cytokine, IL-6 is a potent inducer of inflammatory sequelae, including from COVID-19 (41, 42). The SARS-CoV-2-associated inflammatory cytokine storm, comprising cytokines including IL-6, leads to severe illness and multiple organ dysfunction (13, 17, 35, 43). NF-κB-derived high IL-6 levels are biomarkers of COVID-19 severity and predict mortality; IL-6 is thus a potential target for immunotherapy (13, 17, 41–44). Pulmonary fibrosis in severe COVID-19 cases or as sequelae after COVID-19 are associated with the cytokine storm (45). Because IL-6 is linked to pulmonary fibrosis, for example, during idiopathic pulmonary fibrosis (46), high IL-6 levels during COVID-19 could promote pulmonary fibrosis; this hypothesis would need further investigation. Since auranofin inhibits fibrosis in human hepatic stellate cells (47), auranofin could hypothetically have the same inhibitory effect on SARS-CoV-2-associated pulmonary fibrosis; this hypothesis will need to be further investigated.

## Antiviral Properties of Auranofin

Antiviral actions of auranofin have been an active field of research. For example, auranofin has anti-HIV properties. Protein-protein interactions, involving the viral glycoprotein gp120, are essential for HIV to enter human cells (48). Thioredoxin, or another redox enzyme, reduces gp120 for proper conformation for protein interaction and viral entry (48). Auranofin, by inhibiting thioredoxin reductase, was shown to inhibit HIV infection of cultured cells (48). SARS-CoV-2 also relies on protein-protein interactions, particularly between the viral spike protein and host angiotensin converting enzyme 2 (ACE2), for entry into host cells (35, 49). This interaction was illustrated by the crystal structure of the receptor-binding domain of the spike protein bound to the host ACE2 (50, 51). Cysteines are present in spike and ACE2 proteins and are involved in redox-active disulfide bonds (52). Conformation of both the spike and the ACE2 proteins can thus be subject to the redox milieu (52). A thioredoxin-dependent redox model of spike-ACE2 interaction was proposed and the authors hypothesized that oxidized ACE2 with intact disulfide bonds is necessary for SARS-CoV-2 entry, and that the thioredoxin system, including thioredoxin reductase, would block viral entry *via* reduction of ACE2 (52). However, our understanding of the spike-ACE2 interface is not complete because, in fact, auranofin (an inhibitor of thioredoxin reductase) inhibits the spike-ACE2 interaction at IC_50_ value of 22.2 µM (49, 53, 54). Studies using humanized ACE2 transgenic mice would probably determine the potential of auranofin to block SARS-CoV-2 entry in host cells. Auranofin also inhibits SARS-CoV and SARS-CoV-2 papain-like proteases, (which are important for viral replication) at IC_50_ values of 25.5 µM and 0.75 µM, respectively (49).

Rothan et al. reported that, at 24 hours and at 48 hours after infection, auranofin (4 µM) reduced SARS-CoV-2 RNA by 70% and 85%, respectively, in human cell culture supernatants and by 85% and 95%, respectively, in human cell lysates (33). The EC_50_ at 48 hours after infection was approximately 1.4 µM (33). In cell culture supernatants at 48 hours after infection, auranofin also significantly reduced viral infectivity as determined by plaque assay (33). To explain its inhibitory effect on viral replication, it was hypothesized that auranofin could affect SARS-CoV-2 protein synthesis partially due to its action on the redox milieu *via* inhibition of thioredoxin reductase (33). We recommend to further investigate *in vitro* the potential direct antiviral action of auranofin during SARS-CoV-2 infection to better understand its mechanism of action. Then, we encourage pre-clinical studies in animal models of COVID-19 to validate *in vivo* the direct antiviral property of auranofin. Also, pre-clinical studies will determine if treatment with auranofin for more than 48 hours will be safe for SARS-CoV-2-infected animals. Rothan et al. reported that, not only did auranofin inhibit SARS-CoV-2 replication, it also significantly reduced the expression of key proteins of the inflammatory response (IL-6, NF-κB, TNFα, ILβ) 48 hours after infection of human cells with SARS-CoV-2 (33). The effect of auranofin on IL-6 is particularly marked and can probably be explained by inhibition of the NF-κB pathway. In fact, SARS-CoV-2 infection increased by 200-fold the mRNA expression of IL-6 in human cells compared to mock-infected cells, while auranofin-treated SARS-CoV-2-infected cells had only a 2-fold increase in IL-6 expression compared to mock-infected cells (33). High levels of IL-6 in the COVID-19-associated cytokine storm are linked with disease severity and mortality (17, 41). Therefore, to our point of view, the major advantage of auranofin treatment is its significant reduction of IL-6 expression in SARS-CoV-2-infected cells. Animal models, particularly with humanized ACE2 transgenic mice, could determine if auranofin has the potential to reduce SARS-CoV-2-associated IL-6-derived pathogenicity and decrease COVID-19 immunopathology.

Auranofin also inhibits replication of other viruses, including Zika virus and Venezuelan equine encephalitis virus, as well as chikungunya virus both *in vitro* and in a murine model of infection (55). Apoptosis of auranofin-treated virus-infected cells was proven by the presence of viral genomes and misfolded/incompletely assembled particles in supernatants (55). Selective apoptosis triggered by auranofin is illustrated by its action on the HIV viral reservoir. HIV eradication from the body is compromised by the viral reservoir in long-lived central memory and transitional memory CD4^+^ T cells harboring the retroviral genome (56). *In vitro*, auranofin induces differentiation and death of these CD4^+^ T cell subpopulations constituting the viral reservoir in humans (56). Also, in SIVmac251-infected rhesus macaques, auranofin reduced long-lived central memory and transitional memory CD4^+^ T cells and decreased cell-associated viral DNA (56). The anti-HIV reservoir effects of auranofin were attributed to the lower antioxidant defenses of central memory and transitional memory CD4^+^ T cells (57). The inhibitory effect of auranofin on the redox enzyme thioredoxin reductase increases cellular oxidative stress and promote redox-sensitive apoptosis (27); auranofin therefore has the ability to select those cell populations for apoptosis (57). In addition, human cells also possess mitochondrial thioredoxin reductase and auranofin can, at very low micromolar concentrations, inhibit mitochondrial thioredoxin reductase (58). Consequently, auranofin can induce mitochondrial membrane permeability transition, loss of mitochondrial membrane potential, release of cytochrome c and apoptosis (58).

## Safe Use of Auranofin in Humans

Auranofin has been generally replaced in the treatment of rheumatoid arthritis by more targeted therapies, in part due to its side effects (32). Auranofin is not a harmless drug and treated patients should be monitored (28). Effectively, as a metal-based drug, toxicity issues need to be considered (54). Loose stool and diarrhea commonly occur following oral treatment with auranofin, while rash and proteinuria are less common side effects (28). Fortunately, thrombocytopenia and bone marrow suppression only happen rarely; but long term therapies should be monitored for their potential impact on immune functions (28). Also, auranofin is not recommended in pregnant women and a highly effective birth control should be used (59). In a recent Phase I clinical trial investigating the potential of auranofin for short term therapies, this gold compound was safe and well tolerated (59). Adverse effects were frequent (headache was the most frequently reported), but all adverse effects were mild and resolved without treatment (59). In most cases, anti-COVID-19 treatment with auranofin should be short term (54). The advantages of auranofin treatment in SARS-CoV-2 infections will prevail over potential toxicity for most patients, but we recommend administering auranofin under close medical supervision until safety data are available for COVID-19 patients. Auranofin is FDA-approved and safe in humans and the fear of side effects, which are mostly mild, should not stop its evaluation in clinical trials for the treatment of COVID-19. In fact, auranofin is now in several Phase II clinical trials for its antiviral and antiparasitic properties (27, 33, 59). Auranofin could be tested by adding it to currently accepted therapies with minimal additional risk for COVID-19 patients. If clinical trials prove its safety in COVID-19 patients, auranofin could then be administered outside of hospital settings.

## Discussion

An orally-administered FDA-approved drug with anti-inflammatory and anti-SARS-CoV-2 properties with an acceptable toxicity profile would offer significant benefits for the control of the COVID-19 pandemic. Auranofin, an anti-rheumatic drug, has anti-inflammatory properties *via* its modulation of the NF-κB-IL-6-STAT3 signaling pathway by inhibiting several components upstream and downstream of IL-6 expression (34, 36, 39, 40). Because NF-κB and NF-κB-dependent IL-6 are major actors in the COVID-19-associated cytokine storm and high IL-6 levels worsen prognosis and predicts mortality (13, 17, 35, 41), drugs that can block this pathway are of immediate interest (18–21, 42–44). Auranofin is a potent inhibitor of SARS-CoV-2-induced cytokine expression (including IL-6) in human cells *in vitro* (33). Moreover, unlike monoclonal antibodies specifically targeting IL-6 or its receptor or glucocorticoids such as dexamethasone, auranofin also has a potential direct antiviral action that could be of additional benefit. Effectively, if further investigations *in vitro* and in COVID-19 animal models (such as in humanized ACE2 transgenic mice) validate the potential direct anti-SARS-CoV-2 action of auranofin, it will constitute an additional factor in its favor. Furthermore, by inhibiting thioredoxin reductase, auranofin can induce redox-sensitive apoptotic pathways and promote mitochondrial membrane permeability transition (27, 58). Studies will be needed to determine if auranofin can target for apoptosis SARS-CoV-2-infected cells. Auranofin also has an anti-fibrotic action in human hepatic stellate cells (47), but it is not yet elucidated if auranofin can inhibit pulmonary fibrosis in severe COVID-19 cases or as sequelae after COVID-19.

Auranofin shows a peak plasma gold concentration 1-2 h following oral dosing (27, 59–61). Because of rapid metabolism, auranofin is not detected intact in blood; pharmacokinetic data therefore rely on measurement of plasma gold concentration, where gold mostly binds to serum proteins (27, 59–61). Following 7 days of 6 mg/day auranofin, plasma gold concentration ranged from 0.12 to 0.22 µg/ml (59). A clinical trial (Phase I and II Study of Auranofin in Chronic Lymphocytic Leukemia (CLL); registration number NCT01419691) using 21 mg/day auranofin to treat relapsed leukemia was approved by the FDA (59). At a dose of 21 mg/day auranofin, plasma gold concentration increases proportionally and ranged from 0.42 to 0.77 µg/ml (59). Also, doubling the period of treatment from 7 to 14 days doubled the plasma gold concentration, which ranged from 0.22 to 0.42 µg/ml for 6 mg/day auranofin and from 0.78 to 1.48 µg/ml for 21 mg/day auranofin, respectively (59). Moreover, auranofin has a long terminal half-life (59). Therefore, two weeks after the last treatment, plasma gold concentrations remained high ranging from 0.11 to 0.30 µg/ml for 6 mg/day auranofin and from 0.39 to 1.05 µg/ml for 21 mg/day auranofin, respectively (59). Because gold corresponds to 29% of the mass of auranofin, the molarity of ‘auranofin equivalent’ can be calculated from plasma gold molar concentration. But it is important to remember that auranofin itself, as used for *in vitro* assays, is not present in blood and its metabolites might not have the same efficacy *in vivo*, particularly because they are protein-bound (59–61). With this in mind, ‘auranofin equivalent’ values can be extrapolated from plasma gold concentrations mentioned above (59). After 14 days of treatment with 21 mg/day auranofin, plasma gold concentration reached 1.18 µM to 2.21 µM ‘auranofin equivalent’ and remained between 0.58 µM to 1.5 µM ‘auranofin equivalent’ two weeks after the last treatment (59). The antimicrobial properties of auranofin metabolites bound to serum proteins are unknown and should be further investigated. In other words, it may not be appropriate to assume that plasma gold concentration corresponding to a given ‘auranofin equivalent’ molarity would be as efficacious *in vivo* to inhibit infection as the same molar concentration of auranofin *in vitro*; also, the stability of auranofin in cell culture has not been reported. That being said, it is most likely that the concentration needed to inhibit SARS-CoV-2 papain-like protease (IC_50_ 0.75 µM *in vitro*) and to reduce SARS-CoV-2 replication (EC_50_ 1.4 µM *in vitro*) would be achievable by oral administration of auranofin (33, 49). On the other hand, it is improbable that levels obtained after oral administration of auranofin will reach the concentration needed to inhibit the spike-ACE2 interaction (IC_50_ 22.2 µM *in vitro*) (49, 53, 54). Animal studies, particularly with humanized ACE2 transgenic mice, will better define the anti-SARS-CoV-2 effects of auranofin *in vivo*. Notably, in the *in vitro* study reported by Rothan et al., the 50% cytotoxic concentration for human cells was approximately 5.7 µM, 4 times the EC_50_ for SARS-CoV-2 (33). We recognize that this low margin of safety is a source of concern. Because treatment of COVID-19 would be short term, high doses of auranofin may have an acceptable toxicity profile; but clinical trials in COVID-19 patients are of course necessary to establish the appropriate dose.

Injectable gold compounds are also potential therapies for COVID-19 (49, 53, 54, 62). However, investigations remain to be done to determine if, like auranofin (33), other gold metallodrugs inhibit NF-κB-induced IL-6 expression and SARS-CoV-2 replication. The main advantage of gold compounds administered intramuscularly is achieving high plasma gold levels, up to ten-fold higher than with oral administration of auranofin (60, 61). Therefore, the high plasma concentrations needed to inhibit the spike-ACE2 interaction could potentially be achievable with injectable gold compounds (49); however, this hypothesis should be tested in animal models, particularly with humanized ACE2 transgenic mice. On the other hand, it might not be necessary to inhibit the spike-ACE2 interaction to have anti-SARS-CoV-2 effects and auranofin could present other advantages over injectable gold metallodrugs. Effectively, if further *in vitro* investigations followed by pre-clinical studies in animal models of COVID-19 prove that auranofin can inhibit NF-κB-induced IL-6 expression as well as SARS-CoV-2 replication and SARS-CoV-2 papain-like protease, auranofin could become a valuable addition to available therapies for COVID-19. Promising data obtained from pre-clinical studies would support evaluation of auranofin in clinical trials with COVID-19 patients. Another point in the favor of auranofin is that it is conveniently administered orally and for this reason, can be prescribed to patients recovering at home. If clinical trials prove that auranofin can be safely administered to COVID-19 patients, then auranofin could be prescribed outside of hospital settings. Finally, auranofin is mostly excreted by the enteric route (60, 61). Because only 15% to 30% of an oral dose of auranofin is absorbed, even the anti-rheumatic dose of 6 mg/day for 7 days leads to high gold concentration in feces following oral dosing (59–61). High ACE2 expression in enterocytes of the small intestine was proposed to explain enteric COVID-19 symptoms and sites of tissue damage (35). Therefore, we hypothesize that auranofin can be particularly efficacious to treat SARS-CoV-2 infection in the intestines, but this hypothesis would need further investigation in animal models and, potentially, in clinical trials.

## Concluding Remarks

While information on the action of auranofin during SARS-CoV-2 infection is limited, knowledge of the mechanism of action of auranofin in the treatment of autoimmune and infectious diseases allow to predict that this compound could be a promising candidate in the treatment of COVID-19. Due to the current pandemic, there is a necessity to perform further *in vitro* investigations and conduct pre-clinical studies in SARS-CoV-2-infected animals to evaluate the potential anti-inflammatory and antiviral effects of auranofin during SARS-CoV-2 infection. If pre-clinical studies prove the anti-COVID-19 properties of auranofin, these data will pave the way for clinical trials with SARS-CoV-2-infected patients to repurpose auranofin for the treatment of COVID-19. Further studies could also determine if the inhibitory effect of auranofin on thioredoxin reductase can induce redox-sensitive apoptosis of SARS-CoV-2-infected cells and if its anti-fibrotic action can prevent COVID-19-associated pulmonary fibrosis. Ultimately, this oral therapy could be added to supportive therapies administered to patients under monitoring in hospital settings and could benefit COVID-19 patients with mild to moderate symptoms recovering at home. We therefore join our voices with other researchers (33, 49, 53, 54, 62) to encourage the evaluation in animal models and clinical trials of chrysotherapy with auranofin, or possibly other gold metallodrugs, alone or in combination with other immunomodulatory molecules for the treatment of COVID-19.

## Author Contributions

KS-D wrote the original draft of the manuscript. KS-D and MN reviewed and edited the manuscript. All authors contributed to the article and approved the submitted version.

## Funding

KS-D is supported by the Mitacs Accelerate program. MN and the National Reference Centre for Parasitology are supported by the Public Health Agency of Canada/National Microbiology Laboratory, the Foundation of the McGill University Health Centre, the McGill Interdisciplinary Initiative in Infection and Immunity and the Research Institute of the McGill University Health Centre.

## Conflict of Interest

The authors declare that the research was conducted in the absence of any commercial or financial relationships that could be construed as a potential conflict of interest.

## Publisher’s Note

All claims expressed in this article are solely those of the authors and do not necessarily represent those of their affiliated organizations, or those of the publisher, the editors and the reviewers. Any product that may be evaluated in this article, or claim that may be made by its manufacturer, is not guaranteed or endorsed by the publisher.
